# Impact of Early Intervention for Idiopathic Normal Pressure Hydrocephalus on Long-Term Prognosis in Prodromal Phase

**DOI:** 10.3389/fneur.2022.866352

**Published:** 2022-04-11

**Authors:** Yoshinaga Kajimoto, Masahiro Kameda, Akihiro Kambara, Kenji Kuroda, Shohei Tsuji, Yasutaka Nikaido, Ryuichi Saura, Masahiko Wanibuchi

**Affiliations:** ^1^Department of Neurosurgery, Osaka Medical and Pharmaceutical University, Takatsuki, Japan; ^2^Clinical Department of Rehabilitation, Osaka Medical and Pharmaceutical University, Takatsuki, Japan; ^3^Department of Physical and Rehabilitation Medicine, Osaka Medical and Pharmaceutical University, Takatsuki, Japan

**Keywords:** idiopathic normal pressure hydrocephalus (iNPH), early intervention, CSF shunt, long-term outcome, hydrocephalus surgery, prodromal phase

## Abstract

**Objectives:**

Because the progression of idiopathic normal pressure hydrocephalus (iNPH) is partially irreversible, we hypothesized that early intervention would markedly improve its prognosis. To test this hypothesis, we retrospectively investigated the long-term prognosis of patients with early intervention in the prodromal phase of iNPH.

**Methods:**

We defined the prodromal phase of iNPH as a 3m Timed Up and Go (TUG) of 13.5 s or less and a Mini-Mental State Examination (MMSE) of 24 or more. Of the 83 iNPH patients who underwent shunt surgery at Osaka Medical and Pharmaceutical University Hospital over 3 years from January 2015, 12 prodromal phase cases (73.3 ± 6.2 years, 10 males and 2 females) were included in the study. The iNPH grading scale (INPHGS), MMSE, Frontal Assessment Battery (FAB), intermittent gait disturbance (IGD), social participation status, and development of comorbidities were evaluated over 4 years.

**Results:**

Preoperative MMSE was 27.2 ± 1.5, FAB was 14.1 ± 1.8, TUG was 10.7 ± 1.4 s, and total iNPHGS was 2.8 ± 1.4. At 1, 2, 3, and 4 years postoperatively, total INPHGS improved to 0.8, 0.9, 1.5, and 1.7, respectively, and remained significantly better than preoperatively except at 4 years postoperatively. The MMSE improved slightly to 27.5 after 1 year and then declined by 0.35 per year. After 4 years, the mean MMSE was 26.1, and only one patient had an MMSE below 23. FAB improved to 15.2 after 1 year and then declined slowly at 0.85/year. Ten patients (83%) maintained a high capacity for social participation postoperatively. The preoperative tendency to fall and IGD in 9 (75%) and 8 (67%) patients, respectively, completely disappeared postoperatively, resulting in improved mobility. Shunt malfunction associated with four weight fluctuations and one catheter rupture caused temporary worsening of symptoms, which were recovered by valve re-setting and catheter revision, respectively.

**Conclusion:**

Early intervention in the prodromal phase of iNPH patients maintained good cognitive and mobility function and social participation ability in the long term. The maintenance of long-term cognitive function suggests its preventive effect on dementia. To realize early intervention for iNPH, it is desirable to establish an early diagnosis system for iNPH.

## Introduction

Idiopathic normal pressure hydrocephalus (iNPH), which is a progressive neurological disease resulting in dementia and gait and balance disorders, as well as urinary incontinence, is treatable by cerebrospinal fluid (CSF) shunting (1, 2) demonstrated that the progression of iNPH is partially irreversible manner (3). The reversible part of the progression suggests the efficacy of CSF shunting, while the irreversible part suggests that the earlier the intervention, the better the prognosis. Mild cases have been reported to have a better short-term prognosis (4, 5) and long-term prognosis of cognitive function (6). However, the prognosis of early intervention in patients with iNPH in milder cases, which may be termed the prodromal phase, is unknown.

Many neurodegenerative diseases progress irreversibly, making it difficult to improve their prognosis. In Alzheimer's disease (AD), recent large-scale clinical trials have failed (7–9). Therefore, interventions to control the progression of AD are shifting to earlier stages, i.e., mild cognitive impairment (MCI) (10) and preclinical AD (11, 12). Also in Parkinson's disease (PD), it has been suggested that intervention in the prodromal phase may improve symptom progression (13). However, even though iNPH is also an irreversible neurological disease, there is still no concept of early diagnosis and early intervention.

iNPH develops after a preclinical phase named asymptomatic ventriculomegaly with features of iNPH on MRI (AVIM) (14). Although iNPH should have a prodromal phase as in AD and PD, it has not been defined. Therefore, we defined the prodromal phase of cognitive symptoms in iNPH as an MMSE of 24 or higher, referring to the MMSE cutoff values for AD and MCI (15, 16). It has also been suggested that patients with suspected prodromal symptoms of PD have minimal motor features (MMF) (17). MMF is defined as two or more Parkinsonian signs with a Movement Disorder Society-Sponsored Revision of the Unified Parkinson's Disease Rating Scale (MDS-UPDRS) (18) score of 1, but without sufficient clinical features for a diagnosis of PD (13). The description of a score of 1 for gait in the MDS-UPDRS is “slight: independent walking with minor gait impairment” (18). Since this definition is not quantitative, we defined a 3m-timed up and go test (TUG) of 13.5 s or less as the prodromal phase of gait balance function in iNPH, with a low risk of falling (19). On the other hand, urinary incontinence is not included in the definition of prodromal iNPH because there are many age-related urinary problems such as benign prostatic hyperplasia, overactive bladder, and abdominal stress urinary incontinence.

In this study, we hypothesized that early intervention in patients with a prodromal phase of iNPH (MMSE ≥ 24 and TUG ≤ 13.5 s) would have a very good long-term prognosis, and we tested this hypothesis retrospectively.

## Materials and Methods

### Eligible Patients

The study protocol was approved by the Ethics Committee of Osaka Medical and Pharmaceutical University (No. 2844). We defined the prodromal stage of Idiopathic normal pressure hydrocephalus (iNPH) as a 3m Timed Up and Go (TUG) of 13.5 s or less and a Mini-Mental State Examination (MMSE) of 24 or more. Of the 83 iNPH patients who underwent shunt surgery at Osaka Medical and Pharmaceutical University Hospital over 3 year from January 2015, 14 (17%) were in the prodromal phase. Of these, 12 patients (72.9 ± 5.8 years old, 11 males and 2 females) who could be followed up for more than 4 years were included in the study. The two excluded patients both had symptomatic improvement after shunting but suffered sudden cardiovascular death and terminal cancer around 2 years after surgery. The indications for cerebrospinal fluid (CSF) shunt surgery were following the “Guidelines for management of iNPH (Third edition)” (20). However, we modified the definition of ventricular enlargement to an Evans' index of 0.27 or greater and emphasized the tightness of the higher subarachnoid space rather than ventricular enlargement. In the CSF tap test, the TUG and MMSE and frontal assessment battery (FAB) were assessed. Four patients were treated with ventriculoperitoneal shunting and eight with lumboperitoneal shunting. In all cases, a programmable valve was used and the initial pressure was set by referring to a quick reference table (21, 22). The initial pressure was then reset in five patients (42%).

### Measurement Parameters

Measured parameters including iNPH grading scale (INPHGS) (23, 24), and cognitive function were retrospectively obtained from electronic medical records. Gait and cognitive functions were assessed by physical and speech therapists, respectively. Due to Covid-19, FAB follow-up was discontinued after 3 years. INPHGS was assessed by a neurosurgeon. High-performance activities such as sports, hobbies, and work as indicators of high social participation, development of comorbidities such as dementia and stroke, tendency to fall, and intermittent gait disturbance (IGD) (25) were also recorded for 4 years. Furthermore, we analyzed the process that led to the early diagnosis from the medical history.

### Statistical Analysis

Data are presented as mean values (± standard deviation). Changes in INPHGS, MMSE, and FAB were analyzed using Wilcoxon's signed rank test. A *p*-value < 0.05 was considered statistically significant. Data analysis was performed using JMP Pro 15.1 (SAS Institute Inc., Cary, NC, USA).

## Results

### Change in INPH Grading Scale

**Figure 1:**
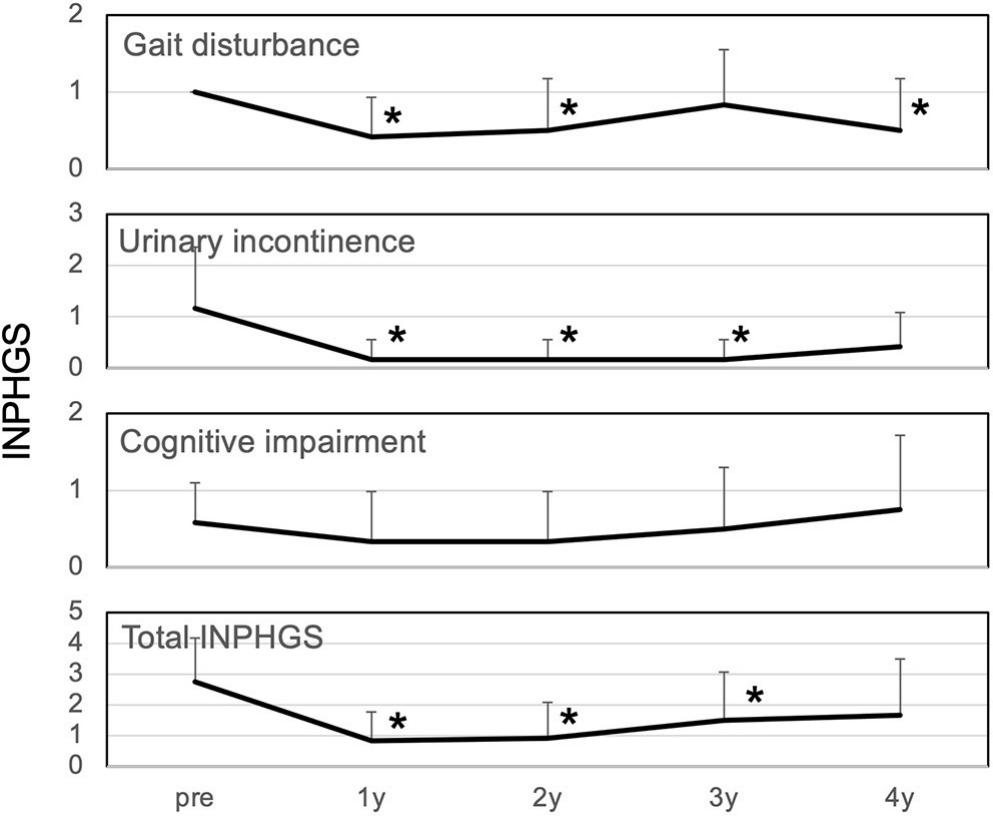
Changes in iNPH grading scale over 4 years. All symptoms improved at 1 year postoperatively. Gait disturbance and urinary incontinence improved significantly at 1, 2, and 4 years postoperatively and at 1, 2, and 3 years postoperatively, respectively. The improvement in cognitive impairment lasted for 1-2 years but returned to the original level at 4 years. Changes in the INPHGS were assessed using Wilcoxon's signed-rank test. INPHGS, idiopathic normal pressure hydrocephalus grading scale. Asterisks indicate significant differences compared to preoperative values (*p* < 0.05).

The total iNPHGS, which was 2.8 preoperatively, continued to improve to 0.8, 0.9, 1.5, and 1.7 at 1, 2, 3, and 4 years postoperatively, respectively (Figure 1). The results were statistically significant up to 3 years after surgery. Among the INPHGS, improvement in gait disturbance was significant at 1, 2, and 4 years postoperatively. The improvement in urinary incontinence was significant at 1, 2, and 3 years postoperatively.

### Changes in Mini-Mental State Examination and Frontal Assessment Battery

**Figure 2:**
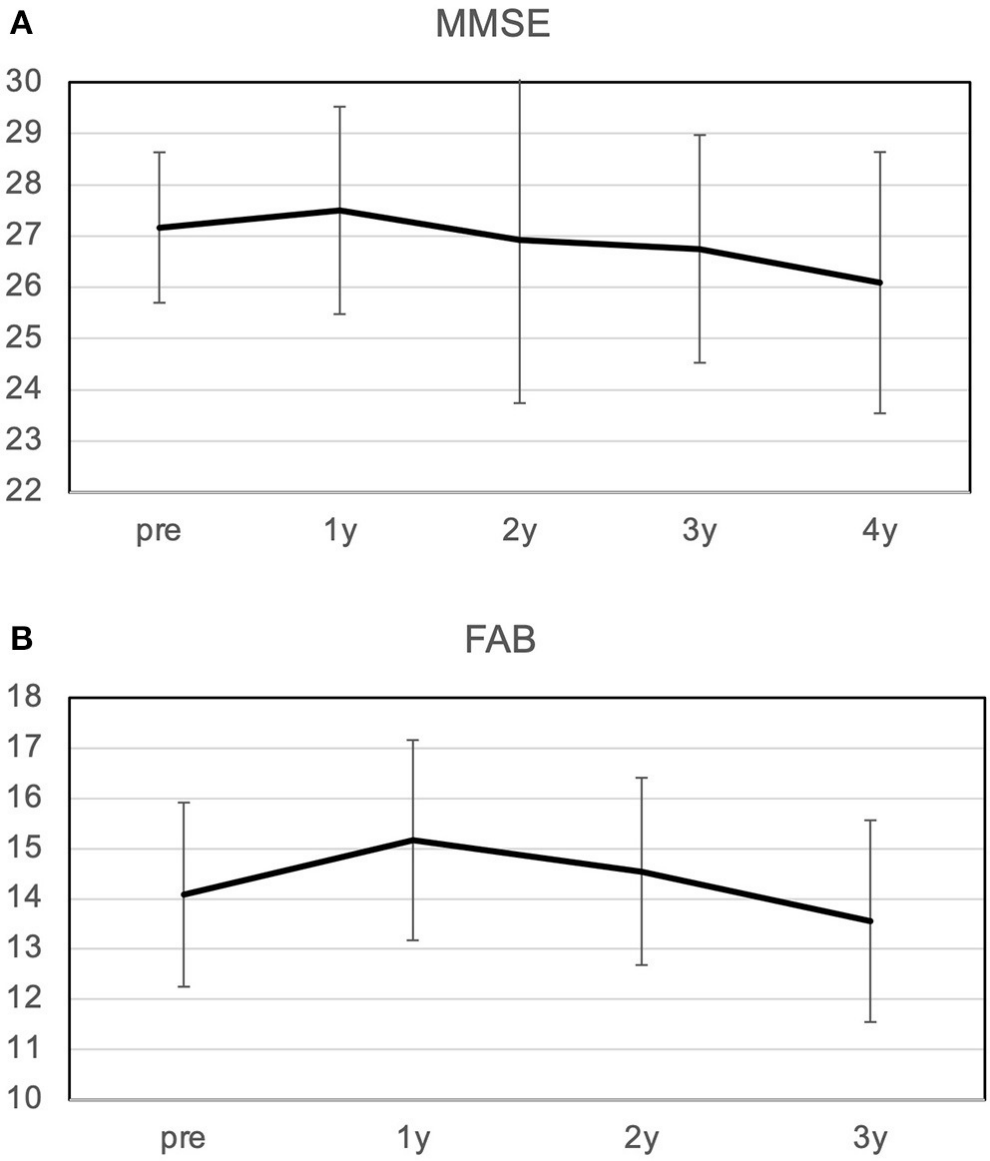
Changes in MMSE **(A)** and FAB **(B)** over 4 years. **(A)** The MMSE improved from 27.2 to 27.5 at 1 year postoperatively and then declined slowly; the average rate of decline over 4 years was 0.26/year. **(B)** FAB improved from 14.1 to 15.2 at 1 year, and then declined slowly at a rate of 0.85/year. MMSE, Mini-Mental State Examination; FAB, Frontal Assessment Battery.

**Figure 3:**
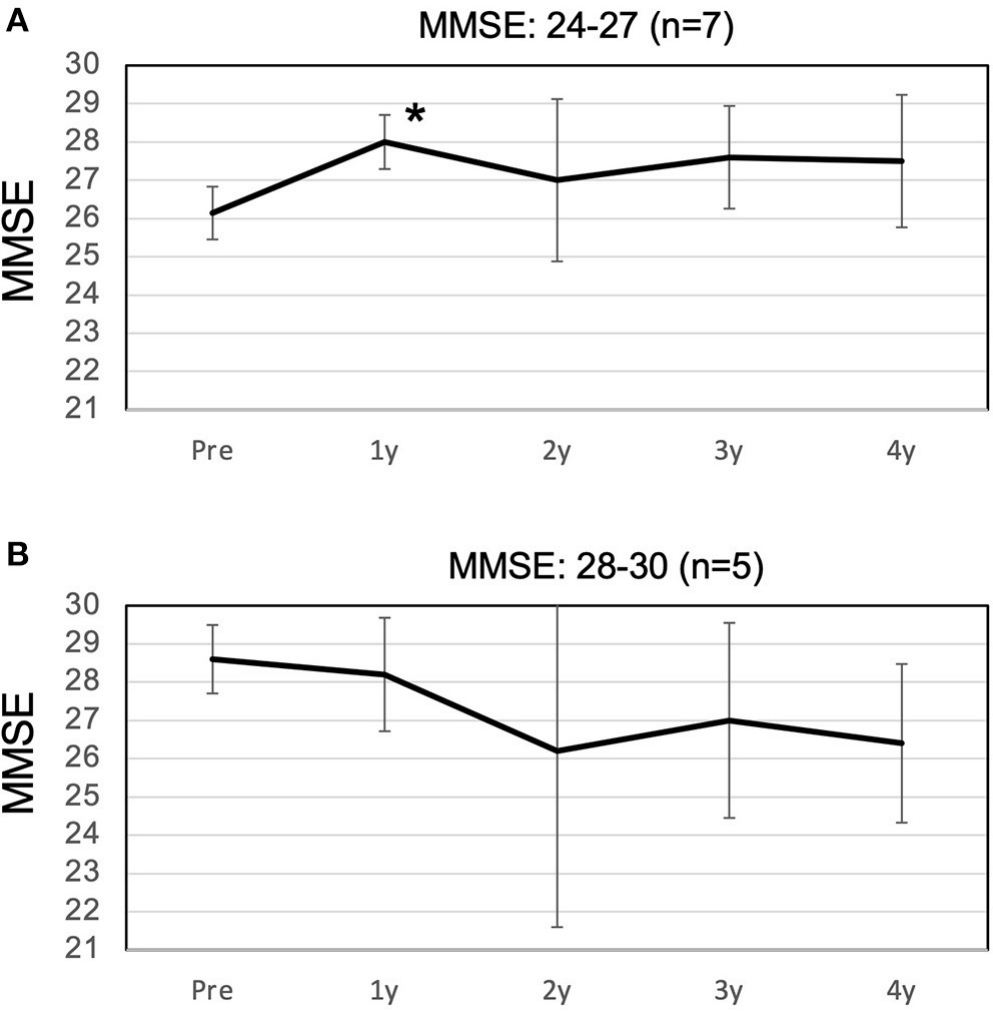
Changes in the high and low MMSE groups over 4 years. **(A)** In the low MMSE group, with a preoperative MMSE of 24-27, the MMSE improved significantly to 28.0 at 1 year postoperatively, with only a slight decline thereafter. **(B)** In the high MMSE group, with a preoperative MMSE of 28-30, the MMSE decreased slowly until 4 years postoperatively. There was no difference between the two groups at 4 years postoperatively. MMSE, Mini-Mental State Examination. Asterisks indicate significant differences compared to preoperative values (*p* < 0.05).

**Figure 4:**
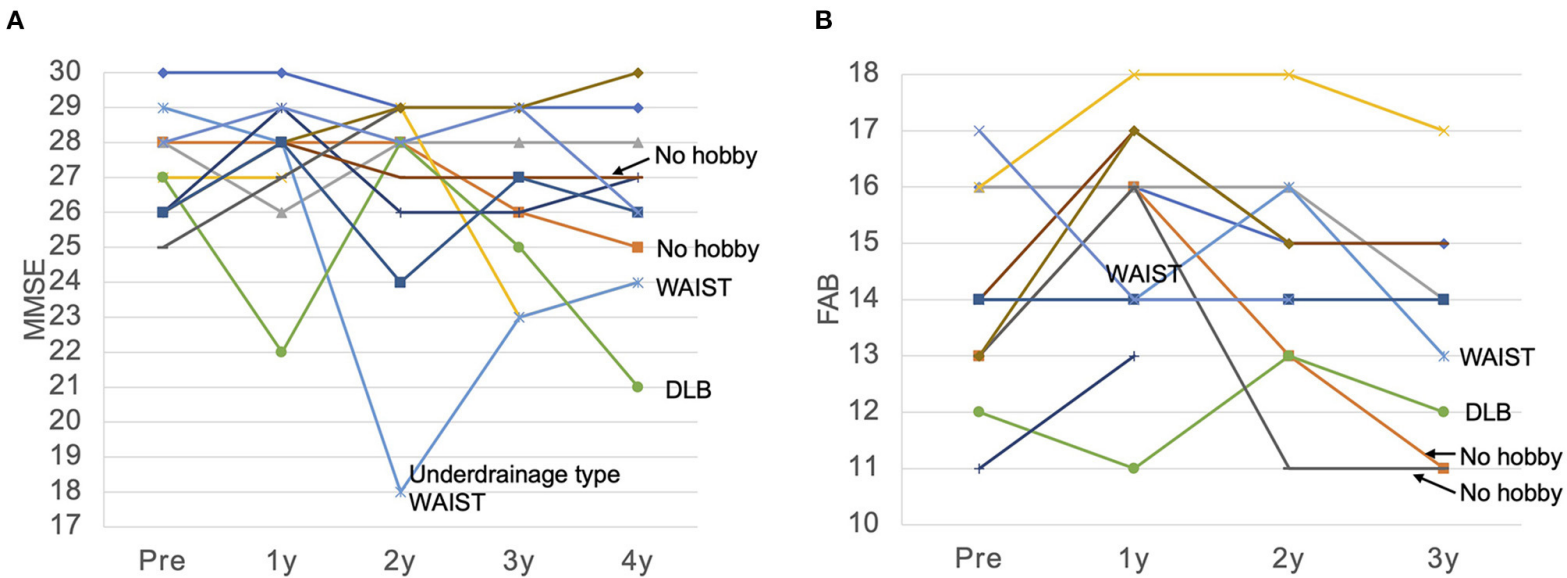
MMSE **(A)** and FAB **(B)** for each patient over 4 years. **(A)** The MMSE remained almost unchanged for 4 years in many patients. MMSE showed a significant decrease in cases of dementia with Lewy bodies (DLB) and functional underdrainage associated with weight gain, which we named weight and abdominal pressure induced shunt trouble (WAIST). **(B)** FAB was also maintained at a high score for 4 years in many patients, but declined significantly in one patient who developed DLB, in two patients with no hobbies and a persistent inactive lifestyle, and in one patient with an underdrainage type of WAIST. MMSE, Mini-Mental State Examination; FAB, Frontal Assessment Battery; WAIST, weight and abdominal pressure induced shunt trouble.

The MMSE improved slightly from 27.2 preoperatively to 27.5 at 1 year postoperatively, and decreased slightly to 26.1 at 4 years postoperatively (Figure 2). The mean MMSE decreased by 1.1 from 27.2 to 26.1 over the 4 years, indicating that the annual rate of decline in MMSE was 0.27/year. Patients with a preoperative MMSE of 24-27 showed a significant improvement in MMSE at 1 year and a gradual decline thereafter. On the other hand, patients with an MMSE of 28-30 also showed cognitive decline over time, and there was no difference between the two groups 4 years after surgery (Figure 3). The mean MMSE at 4 years was 26.1, and only one patient had an MMSE below 23 (Figure 4). This patient had fluctuating symptoms, and 123I-IMP SPECT showed relative cerebral hypoperfusion in the temporoparietal and occipital lobes, suggesting the development of dementia with Lewy bodies. One patient (8%) progressed to dementia defined by an MMSE of 23 or less over 4 years, suggesting an annual conversion rate of 2%/year.

The FAB improved from 14.1 to 15.2 after 1 year and then declined slowly at 0.8/year. Ten patients who originally had hobbies or jobs resumed high-performance social participation after surgery, including running a company, golf, gym, Japanese harp master, pilgrimage, blogging, and travel, and were able to maintain these activities for a long time (Table 1; Figure 4). As a result, their FAB at 3 years postoperatively was maintained at 14.3. On the other hand, two patients originally had no hobbies and continued to live inactive after surgery. They improved their FAB from 13 to 16 after 1 year, and the average annual rate of decline over the next 2 years was 2.5/year. As a result, the FAB rapidly decreased to 11 at 3 years after surgery (Figure 4B). In the case of the patient whose MMSE suddenly dropped to 18 2 years after surgery, the cause was functional underdrainage, as described below (Figure 4A).

**Table 1:** Clinical characteristics and postoperative course of 12 patients with prodromal phase iNPH.

**No**.	**Age**	**Sex**	**OP**	**Fall count**	**IGD**	**TUG (s)**	**Diagnostic trigger**	**High performance activity (duration)**	**MMSE/FAB preOP**	**MMSE/FAB after 1 year**	**MMSE/FAB after 4/3 years**	**Antidepressant preOP/postOP**	**Antidementia drug preOP/postOP**
1	65	M	VP	0	−	10.8	AVIM Following	Bamboo flute (4y-)	30/16	30/16	29/15	Aripiprazole/none	None/none
2	63	M	LP	0	−	11.3	MRI for dementia Dx	none	28/13	28/16	25/11	Aripiprazole/none	None/none
3	74	M	VP	4	+	8.0	CT at fall	President(4y-), Golf(2y)	28/16	26/16	28/14	None/none	None/none
4	72	M	VP	1	+	9.9	AVIM Following	President(2y), Golf(4y-)	27/16	27/18	24/17	None/none	None/Rivastigmine
5	86	M	VP	2	+	11.6	MRI for dementia Dx	Karaoke(4y-)	29/14	28/14	24/13	None/none	None/none
6	77	M	LP	1	−	11.2	MRI for dementia Dx	Pilgrimage(1y)	27/12	22/11	21/12	None/none	None/donepezil
7	71	M	LP	1	+	8.4	MRI for dementia Dx	Gym(4y-)	26/11	29/13	27/NA	Maprotiline/none	None/none
8	77	M	LP	1	+	10.7	MRI for PD Dx	Flower Photography Blog(4y-)	26/14	28/17	27/15	None/none	None/none
9	68	M	LP	1	+	13.2	CT at fall	none	25/13	27/16	29/11	None/none	None/none
10	75	M	LP	2	+	11.5	CT at fall	Golf(4y-), Travel(4y-)	26/13	28/17	30/NA	None/none	None/none
11	74	F	LP	1	+	10.4	AVIM Following	Japanese Harp Master(4y-)	26/14	28/14	26/14	None/none	Donepezil/none
12	78	F	LP	0	−	11.9	MRI for dementia Dx	Chorus(3y), Gym(3y)	28/17	29/14	26/NA	None/none	none/none

*Fall count and IGD represent the number of falls and the presence of intermittent gait disturbance (IGD) before surgery. The 4y- in duration means more than 4 years*.

*INPHGS, iNPH grading scale; IGD, intermittent gait disturbance; MMSE, mini-mental state examination; FAB, frontal assessment battery; LP, lumbo-peritoneal; VP, ventriculo-peritoneal; AVIM, asymptomatic ventriculomegaly with features of iNPH on MRI; PD, Parkinson's disease; Dx, diagnosis*.

Antidementia drugs and antidepressants may affect the MMSE. Six of the 12 patients in this study did not take any of these drugs during the study. Three patients had taken antidepressants preoperatively, and one had taken donepezil, but these were discontinued within the first year postoperatively due to improvement in symptoms. Antidementia drugs were used postoperatively in only two patients with progressive memory impairment after shunt surgery.

### Shunt Malfunction

Mechanical shunt malfunction developed in one patient. Three years postoperatively, the symptoms worsened with delayed rupture of the spinal catheter, but fully recovered with shunt revision. On the other hand, non-mechanical shunt malfunction occurred four times in three patients. There were three cases of underdrainage associated with weight gain, which we named underdrainage type weight and abdominal pressure induced shunt trouble (WAIST). In all cases, valve resetting to increase shunt flow improved symptoms immediately. However, in patients with advanced dementia due to delayed detection of shunt malfunction, recovery of MMSE was limited (Figure 4A). One patient had asymptomatic chronic subdural hematoma with 10 kg weight loss. We have named this phenomenon overdrainage type WAIST. Valve resetting to lower the shunt flow rate made the hematoma disappear after 1 month.

### Improvement in a Tendency to Fall and Intermittent Gait Disturbance

Before surgery, 9 (75%) and 8 (67%) patients tended to fall and intermittent gait disturbance (IGD), respectively (Table 1). However, these completely disappeared after the surgery, resulting in the elimination of fall anxiety and a marked improvement in the patients' mobility.

### Processes Leading to Early Diagnosis

Three patterns in the diagnostic process that led to early diagnosis (Table 1). The first is detection during the differential diagnosis of dementia or Parkinson's disease which was found in six cases (50%). The second is when it is detected by head CT at the time of a fall, which was found in three cases (25%). The third pattern was that the patient had previously been diagnosed with asymptomatic ventriculomegaly with features of idiopathic normal pressure hydrocephalus on MRI (AVIM) and iNPH developed during follow-up, which occurred in three cases (25%).

## Discussion

### Long-Term Outcomes of Interventions in the Prodromal Phase

This study demonstrates that early intervention in patients with idiopathic normal pressure hydrocephalus (iNPH) in the prodromal phase improves the long-term functional outcome of gait, cognition, and urination. Symptoms of gait and urinary dysfunction were significantly improved. On the other hand, early intervention maintained good cognitive function over the long term, but there was no significant difference. The reason for this non-significant difference is thought to be the ceiling effect.

The rate of decline in the Mini-Mental State Examination (MMSE) was as low as 0.27/year, and the conversion rate to dementia was as low as 2%/year. Peterson et al. reported that the rate of decline of MMSE in mild cognitive impairment (MCI) was 1.0/year (10). The rate of decline in MMSE in this study is only one quarter of that reported by them. Therefore, early intervention in prodromal phase iNPH has the potential to reduce the decline in cognitive function over time. It is also known that the annual conversion rate of MCI to dementia is 10-20% (26, 27). The conversion rate to dementia of 2%/year in this study is considerably lower than this.

The study also included five patients with MMSE scores in the normal range of 28 or higher, which may have been associated with a lower rate of cognitive decline. However, there was no difference in MMSE scores at 4 years between the high MMSE group with an MMSE of 28 or higher and the low MMSE group with an MMSE of 24 to 27 (Figure 3). Therefore, it can be concluded that the inclusion of patients with MMSE scores in the normal range of 28 or higher has no effect on the prognosis of cognitive function. Despite the disadvantage of the small number of cases, it suggests that early shunt surgery has a preventive effect on conversion to dementia.

Antidepressants and antidementia drugs may affect the MMSE. In this study, only 2 out of 12 patients took antidementia drugs postoperatively. This fact indicates that the influence of antidementia drugs on this study is limited. On the other hand, three patients had taken antidepressants preoperatively but discontinued them postoperatively. This fact may have contributed to the improvement of frontal lobe function.

The prognosis for cognitive function in shunted iNPH patients has been thought to be poor (28, 29). Koivisto et al. (29) reported that even among patients who responded to shunting, 46% developed dementia during a mean postoperative follow-up of 4.8 years. In the present study, only one patient (8%) converted to dementia over the 4-year period, indicating that early intervention not only has the effect of slowing the rate of cognitive decline but also of preventing conversion to dementia.

### Criticism and Limitation of This Study

It may be criticized that this study treated iNPH in the preclinical phase rather than the prodromal phase because of the favorable long-term prognosis. However, we believe that the patients in this study had minor symptoms of iNPH for the following three reasons. First, there was a significant improvement in gait and urinary dysfunction in iNPH grading scale (INPHGS). Second, there was a reversible progression of symptoms with shunt malfunction. Third, the preoperative tendency to fall and intermittent gait disturbance (IGD) (25), which we also call hydrocephalic intermittent claudication (HIC), is caused by balance disturbance seen in long-distance walking, disappeared after CSF shunting. The present study is a retrospective study with few cases, which limits the evidence. Therefore, prospective cohort studies and randomized controlled studies are desirable to prove the effectiveness of early intervention for iNPH at a high level of evidence.

### Definition of Prodromal Phase of iNPH

The prodromal phase of iNPH in this study is defined as the prodromal phase of MCI in Alzheimer's disease (AD) (15, 16), minimal motor features (MMF) in Parkinson's disease (PD) (13, 17, 18), and the cutoff value of the 3m-timed up and go test (TUG) in fall risk determination (19) were used as a reference for definition. Therefore, we believe that the definition of the prodromal phase of iNPH is valid currently. However, further discussion on its validity, cut-off values, and the choice of assessment methods for cognition, gait, and balance is necessary.

### Shunt Malfunction

In the present study, functional and non-mechanical shunt malfunctions caused by weight gain or loss occurred four times more frequently than mechanical shunt malfunctions. The cause of non-mechanical shunt Malfunctions is mainly due to the increase in intra-abdominal pressure with weight gain, which results in an increase in intracranial pressure outside the appropriate therapeutic window (in print). Similar shunt malfunctions have been reported rarely in pregnant women (30). However, its high frequency in iNPH patients may be due to the narrow therapeutic window for valve setting pressure in iNPH patients. When a shunt Malfunction is found in a patient with iNPH, weight gain should be checked. If there is weight gain, lowering the valve setting is likely to improve the worsening symptoms.

### Frontal Lobe Function and Social Participation

**Figure 5:**
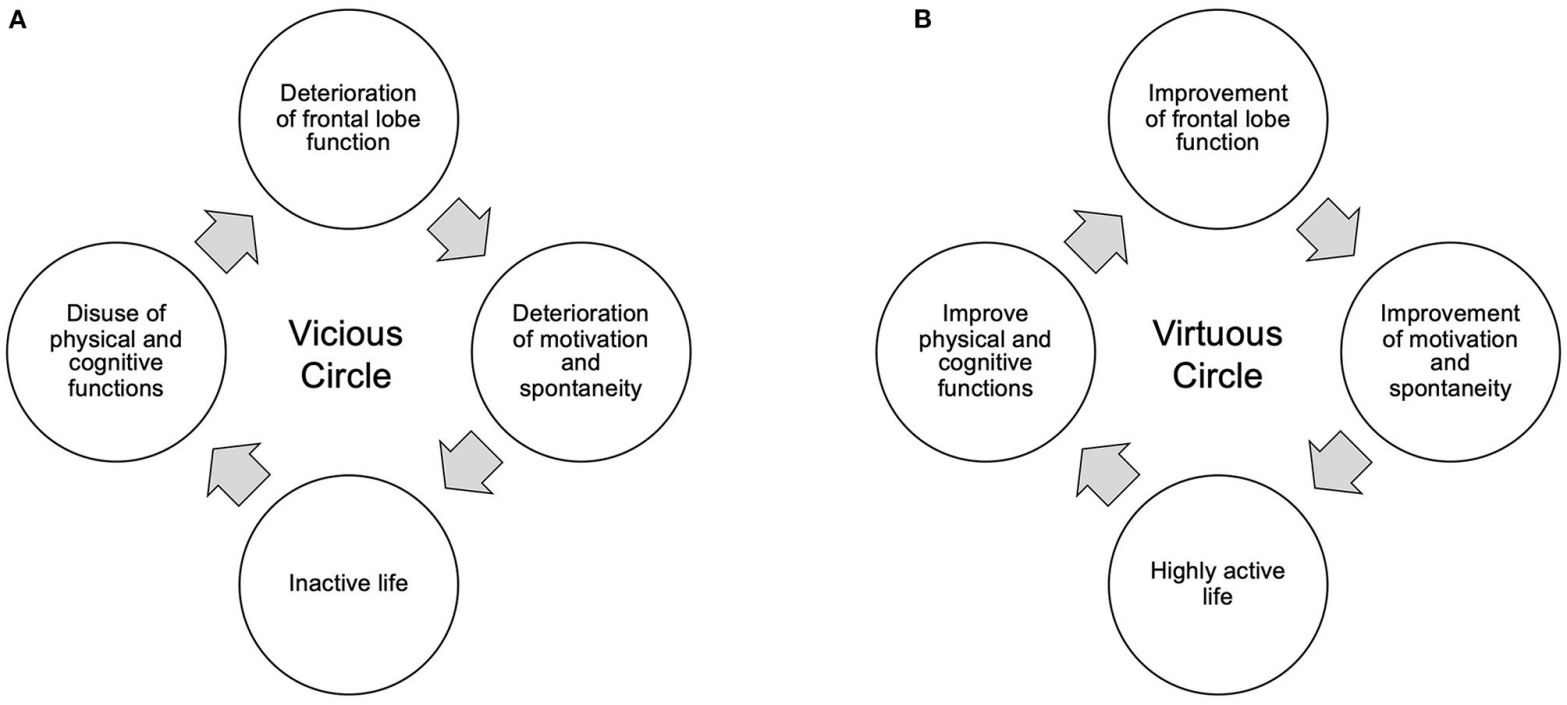
Vicious **(A)** and virtuous **(B)** circles between frontal lobe function and activity of life. **(A)** Deterioration of the patient's frontal lobe function reduces motivation, such as spontaneity, and leads the patient to a more inactive life. As a result, cognitive and physical functions decline due to disuse, leading to a vicious cycle of further decline in frontal lobe function. **(B)** Maintaining and improving frontal lobe function maintains and improves motivation, including spontaneity, and restores an active lifestyle. As a result, cognitive and physical functions enter a virtuous cycle of improvement.

Patients with moderate iNPH are prone to a vicious cycle in which, even if gait function improves, motivation and spontaneity continue to decline, resulting in an inactive lifestyle, disuse of cognitive and motor functions, and more inactive living (Figure 5A). On the other hand, in the present study, many patients were able to resume or maintain a high level of social participation through work or hobbies. This suggests a virtuous cycle of activation of physical and mental activities through a high level of social participation (Figure 5B; Table 1). Even though MMSE did not significantly improve postoperatively due to ceiling effect, gait significantly improved and frontal lobe function was maintained. This is thought to have enabled the resumption and maintenance of social participation, which in turn prevented the decline in cognitive function.

It is thought that the frontal lobe functions, particularly the prefrontal cortex, are responsible for this motivation to participate in society (31, 32). A variety of executive functions such as planning, decision making, and short-term memory are essential for maintaining a high level of social participation. Its frontal lobe function can be measured by the Frontal Assessment Battery (FAB). In the present study, the FAB was maintained in the normal range in patients who were able to participate in society, while it decreased in patients who were unable to maintain social participation. In the two patients who had originally had no hobbies and poor social participation, the FAB improved to 16 1 year after surgery, and then rapidly declined to 11. These facts suggest not only that high frontal lobe function is important for social participation, but also that social participation through hobbies and work is essential for maintaining that frontal lobe function.

### Medical System Leading to the Early Diagnosis of iNPH

**Figure 6:**
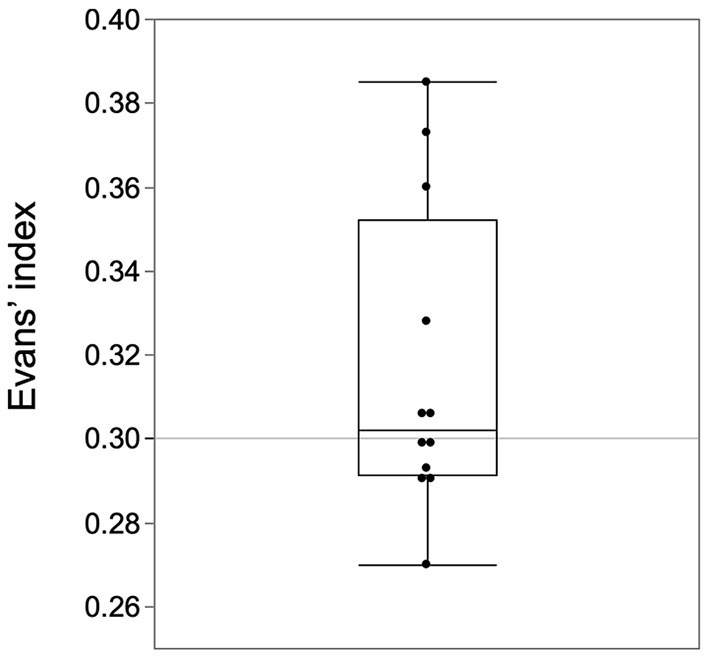
Half of the early cases had an Evans' Index of <0.3. The Evans index of 0.3 or higher in the guidelines should be revised for early-stage cases.

The three diagnostic routes that led to early diagnosis had the following problems. One problem common to all the routes is the cutoff value of the Evans' Index. Evans' Index of 0.3 or higher is commonly used as a diagnostic criterion for iNPH (20). However, in the early cases of our study, half of the patients would be excluded if this criterion were used (Figure 6). The distribution in the histogram of the Evans' index of iNPH patients reported in the SINPHONI study has the third highest frequency of 0.30-0.32 (1). In this Evans' index distribution, there is an incongruous absence of frequencies below 0.30. This suggests that there may be iNPH patients with Evans' index below 0.30. There are two ways to avoid this problem. First, the cutoff value of 0.3 for the Evans' Index should be reviewed. Second, the tightness of the higher subarachnoid space should be the first focus of attention rather than ventricular enlargement.

The problem with detection at the time of fall is that minor gait disturbances in early-stage patients can easily be missed. TUG of <13.5 s in this study should have a low risk of falling, but many patients did fall. This discrepancy can be explained by gait disturbance, which we have named intermittent gait disturbance (IGD) (25). Patients with IGD can walk almost normally in the office. However, they often become propulsed by postural instability after long-distance walking (25). To avoid this oversight, patients who have fallen should be asked if long-distance walking causes gait instability.

The problem with early diagnosis from AVIM is that this method has not been generalized. When CT and MRI scans of elderly patients are performed and DESH findings are found incidentally, the patient should be provided with the following information. First, there is a 17% annual risk of conversion to iNPH (33). Second, since the symptoms of iNPH are partly irreversible and progressive, a physician familiar with iNPH care should be consulted if the disease develops.

## Data Availability Statement

The data that support the findings of this study are available from the corresponding author upon reasonable request.

## Ethics Statement

The studies involving human participants were reviewed and approved by Ethics Committee of Osaka Medical and Pharmaceutical University (No. 2844). Written informed consent for participation was not required for this study in accordance with the national legislation and the institutional requirements.

## Author Contributions

YK, MK, and MW made substantial contributions to the conception and design of the study. KK, YK, ST, YN, and RS collected data regarding the participants and task performance. YK and MK analyzed the data. YK wrote the manuscript. All authors read and approved the submitted version.

## Conflict of Interest

The authors declare that the research was conducted in the absence of any commercial or financial relationships that could be construed as a potential conflict of interest.

## Publisher's Note

All claims expressed in this article are solely those of the authors and do not necessarily represent those of their affiliated organizations, or those of the publisher, the editors and the reviewers. Any product that may be evaluated in this article, or claim that may be made by its manufacturer, is not guaranteed or endorsed by the publisher.
